# Clinical Evaluation of Three Commercial RT-PCR Kits for Routine COVID-19 Diagnosis

**DOI:** 10.3390/pathogens11111389

**Published:** 2022-11-21

**Authors:** Rifat Tasnim Juthi, Saiful Arefeen Sazed, Md Fahad Zamil, Mohammad Shafiul Alam

**Affiliations:** International Centre for Diarrhoeal Disease Research, Bangladesh (icddr,b), Dhaka 1212, Bangladesh

**Keywords:** SARS-CoV-2, RT-PCR, COVID-19, real time PCR, diagnostics, RT-PCR kit, real time RT-PCR

## Abstract

Amongst the multiple ways to diagnose coronavirus disease-2019 (COVID-19), reverse transcription polymerase chain reaction (RT-PCR) remains the reference gold standard, providing fast and accurate results. This study evaluated and compared the performance of three commercially available COVID-19 RT-PCR kits-Aridia^®^ COVID-19 Real-Time PCR Test (CTK Biotech, Inc., Poway, CA, USA), Novel Coronavirus (2019-nCoV) Nucleic Acid Detection Kit (Sansure Biotech Inc., Changsha, China) and Allplex^TM^ 2019-nCoV assay (Seegene Inc., Seoul, Republic of Korea) for the detection of severe acute respiratory syndrome coronavirus 2 (SARS-CoV-2). A total of 326 clinically suspected patients were enrolled for the study, and among them, 209 were diagnosed as positive and 117 as negative when tested with the reference method, US CDC 2019-Novel Coronavirus (2019-nCoV) Real Time RT-PCR Diagnostic Panel. The Aridia^®^ kit showed total agreement with the reference test, with a sensitivity of 100% (95% CI: 98.25% to 100.0%) and a specificity of 100% (96.90% to 100.00%). The Allplex^TM^ kit also showed 100% specificity (95% CI: 96.90% to 100.00%), but a lower sensitivity (98.09%, 95% CI: 95.17% to 99.48%). Among the three kits, the Novel Coronavirus (2019-nCoV) Nucleic Acid Detection Kit showed the worst performance, with a sensitivity of 98.6% (95% CI: 95.9% to 99.7%) and a specificity of 95.73, 95% (CI: 90.31% to 98.60%). While all these kits conform to the requirement for routine molecular diagnosis with high performances, the Aridia^®^ COVID-19 Real-Time PCR Test showed the best performance among the three kits.

## 1. Introduction

COVID-19 has already caused more than 6.5 million deaths and over 625 million cases all over the world (accessed on 21 October 2022) [1]. For fast diagnosis of the disease, there are numerous rapid tests commercially available in the market [2]. Among other diagnostic methods reverse transcription polymerase chain reaction (RT-PCR), Enzyme-linked Immuno Sorbent Assay (ELISA), radiographic analysis (X-Ray, CT-scan), next-generation sequencing (NGS), and whole genome sequencing (WGS) are currently available [3,4,5,6]. Of these, the most common and accurate method to diagnose COVID-19 is quantitative (real-time) RT-PCR analysis of viral RNA [7]. The most used samples for this analysis are nasal, nasopharyngeal, oropharyngeal or naso-oropharyngeal derived, collected with cotton swabs and then transferred into viral transport medium (VTM) [8]. Viral RNA is then extracted from the VTM and subjected to RT-PCR analysis that provides the SARS-CoV-2 status of the sample. 

There are more than 350 commercially available RT-PCR kits that offer highly sensitive and more specific platforms for the diagnosis of COVID-19 [9]. Several commercially available RT-PCR kits for COVID-19 have obtained authorization for emergency use by various authorities such as World Health Organization (WHO) and the United States food and drug administration (US-FDA). Up to recently, twenty-nine kits have obtained US-FDA Emergency Use Authorization (EUA) [9]. Multiple other kits are also available commercially, but a thorough investigation of their performance is warranted so that the most accurate result possible can be obtained at the patient level.

Coronavirus RNA contains several gene targets for molecular detection and, by the amplification of any one or more target genes by RT-PCR, the presence or absence of the virus that causes COVID-19 can be determined. Some of the most commonly amplified genes include the nucleocapsid (N) gene, envelope (E) gene, RNA-dependent RNA polymerase (RdRP), open reading frame 1ab (ORF1ab), N1 and N2 regions of N gene, etc. [10]. Different manufacturers choose different sets of genes for their RT-PCR kits [11]. The different RT-PCR kits generally contain primers, probes, reverse transcriptase enzymes, PCR buffers, RNase-free water, etc. These components come in different formats and containers, and the user typically has to mix them to prepare a master mix for PCR analysis [4]. Some manufacturers offer more user-friendly kits that include a single tube master mix, and the analysts just have to add the RNA template, which minimizes sample handling errors and setup time [12]. 

In this study, we evaluated and compared the diagnostic efficacy of these three commercially available RT-PCR kits-Aridia^®^ COVID-19 Real-Time PCR Test (CTK Biotech, Inc., Poway, CA, USA), Novel Coronavirus (2019-nCoV) Nucleic Acid Detection Kit (Sansure Biotech Inc., Changsha, China) and Allplex^TM^ 2019-nCoV assay (Seegene Inc., Seoul, Republic of Korea) for the purpose of detecting SARS-CoV-2, because these kits are widely used in the diagnostic laboratories of many countries of the world, including ours.

## 2. Materials and Methods

### 2.1. Study Population and Ethical Consideration 

The participants of this study were from Dhaka city, Bangladesh, and were symptomatic at the time of sample collection. Patients coming to the diagnostic facility of the International Center for Diarrhoeal Disease Research Bangladesh (icddr,b) in Dhaka, Bangladesh for a COVID-19 RT-PCR test between 3rd February and 8th August of 2021 were enrolled in the study when most of the circulating variants were beta variant followed by the delta variant. The study was conducted with 326 patients showing fever, cough, sore throats and any other COVID-19 symptoms selected and enrolled for testing by healthcare professionals during that time. This study was approved by the institutional Ethical Review Committee (ERC) of icddr,b. Written informed consent was obtained from the adult participants. For children below 11 years, written informed consent was obtained from the parent or legal guardian. In the case of children between 11 and 17 years of age, verbal assent was also obtained in addition to written consent from the parent or legal guardian.

### 2.2. Specimen Collection

During the specimen collection session, nasopharyngeal as well as oropharyngeal swabs were collected from the enrolled participants in 3 mL of VTM by expert healthcare professionals to perform the gold standard RT-PCR assay.

### 2.3. COVID-19 Diagnosis by RT-PCR

RT-PCR amplification of RNA extracted from naso-oropharyngeal swabs collected in VTM from prospective patients was carried out in the Molecular Diagnostic Laboratory, which is an ISO 15189 and ISO 15190 accredited facility. 

The naso-oropharyngeal samples were extracted using MagMAX™ Viral/Pathogen II nucleic acid isolation kit with a King Fisher Flex high-throughput automated extraction system (Thermo Fisher Scientific, Waltham, MA, USA).

For the reference assay, N1 and N2 gene targets with RNaseP as human internal control were amplified using primer probes suggested by CDC (Center for Disease Control). In brief, TaqPath™ 1-Step RT-qPCR mastermix (Thermo Fisher Scientific, Waltham, MA, USA) was used in ABI 7500 Fast DX instrument (Thermo Fisher Scientific, Waltham, MA, USA) in a 20 μL reaction mix containing 5 μL of the template. The thermocycler conditions consist of a step of reverse transcription (10 min/55 °C), an initial denaturation (1 min/95 °C), and subsequent 45 cycles of denaturation (10 s/95 °C) and annealing/elongation (30 s/55 °C) [7,13]. This assay was considered as a reference RT-PCR assay comprising N1 and N2 CDC primers for COVID-19.

### 2.4. Aridia^®^ COVID-19 Real-Time PCR Test

CTK’s Aridia^®^ COVID-19 Real-Time PCR Test packaging contains a COVID-19 PCR mix, COVID-19 positive and negative controls and PCR-grade water is also provided to reconstitute the lyophilized components. The COVID-19 PCR mix contains all real-time PCR components including DNA polymerase, reverse transcriptase, primers, probes, and dNTPs. For the RT-PCR, 15 µL of PCR mix and 5 µL of RNA sample template, positive control and negative control were mixed to target the ORF1ab, N, E, and a housekeeping gene RNase P. PCR was performed according to the manufacturer’s instruction. A Bio-Rad CFX Opus96 Real-Time PCR System was used for the amplification and the thermocycler was programmed as followed: reverse transcription (10min, 50 °C, one cycle), (2 min, 95 °C, one cycle), denaturation (5 s, 95 °C, 45 cycles) and finally, annealing/extension (20 s, 60 °C, 45 cycles). At the extension step, fluorescence data are collected through HEX, JOE or VIC (ORF1ab), FAM (N gene), Cy5 (E gene), and ROX (RNase P) channels by Bio-Rad CFX Maestro Software Version 2.2. When N, ORF1ab and E genes showed cycle threshold (Ct) < 40, samples were considered positive and when ≥40, samples were considered negative for COVID-19. 

### 2.5. Allplex^TM^ 2019-nCoV Assay (Seegene Inc.)

Components of Seegene’s Allplex^TM^ 2019-nCoV assay kit (ref.: RP10252W, Lot: RP5022A01) are slightly different from Aridia^®^. This kit contains 2019-nCoV MOM, real-time one-step enzyme, 5X real-time one-step buffer and RNase-free water. For preparing the master mix, 2 µL of real-time one-step enzyme and 5 µL of each of the other three components are mixed and 8 µL of RNA template is added for RT-PCR. For amplification, the same instrument was used as the Aridia^®^ test kit; the thermocycler protocol was as follows: reverse transcription (20 min, 50 °C, one cycle), initial denaturation (15 min, 95 °C, one cycle), denaturation (15 s, 94 °C, 45 cycles) and finally, annealing (30 s, 58 °C, 45 cycles). Four fluorophores: Quasar 670, FAM, Cal Red 610 and HEX are selected for the analytes: N gene, E gene, RdRp gene and internal control respectively; the same software recorded the fluorescence data. The Ct value of internal control must be <40, otherwise, the test will be considered invalid and that particular sample should be retested for clarification. If the Ct value is <40 for three other genes, or only for the N gene, the sample is considered COVID-19 positive [14]. 

### 2.6. Novel Coronavirus (2019-nCoV) Nucleic Acid Detection Kit (Sansure Biotech)

Sansure Biotech’s Novel Coronavirus (2019-nCoV) Nucleic Acid Detection Kit (Ref: S3102E, Lot: 2022001-2) for 24 tests contains 2019-nCoV-PCR Mix, 2019-nCoV-PCR-enzyme mix, 2019-nCoV-PCR-positive control and 2019-nCoV-PCR-negative control. As per the manufacturer’s manual, the volume of the reaction mix is 50 µL (30 µL PCR-master mix and 20 µL RNA template) but in this study, maintaining the recommended proportion, 15 µL reaction mix was prepared (9 µL PCR-master mix and 6 µL RNA template) [15]. Cycle parameters were as follows: reverse transcription (50 °C, 30 min, one cycle), cDNA denaturation (95 °C, 1 min, one cycle), denaturation (95 °C, 15 s, 45 cycles), annealing, extension and fluorescence collection (60 °C, 30 s, 45 cycles) and finally device cooling (25 °C, 10 s, one cycle). FAM(ORF1ab), ROX(N) and CY5(IC) channels were selected for signal receiving. Just like the Allplex^TM^ kit, the internal control gene’s Ct value must be <40 to confirm the presence of RNA, otherwise, the samples were retested. To consider a sample COVID-19 positive, the Ct value of the N gene and/or ORF1ab gene must be <40 [15]. 

### 2.7. Lower Limit of Detection (LLOD), Linearity and Assay Reproducibility

To detect the analytical sensitivity of these three kits, the NGS sequence verified SARS-CoV-2 Plasmid Controls with 200,000 copies/µL (Research Use Only) was diluted sequentially to obtain a set of 100,000 copies/µL to 0.01 copies/µL. For each kit, the RT-PCR was performed in triplicates using similar conditions for each one to detect the LLOD. A standard curve was also generated for each kit and the assay was repeated to verify its linearity and reproducibility. The variation was determined in terms of the Coefficient of Variation, CV (the ratio of the standard deviation, SD to the mean) for intra and inter-assay [16].

### 2.8. Data Analysis

For statistical analysis and graphical representation of the data, GraphPad Prism 8.0.2 (GraphPad Software, Inc., San Diego, CA, USA) was used and the diagnostic sensitivity, specificity, positive predictive value (PPV), negative predictive value (NPV), positive and negative likelihood ratio with respective confidence interval were calculated. MedCalc statistical software (V.20.013) (MedCalc Software, Ostend, Belgium) was also used for building Bland–Altman plots. The Cohen’s kappa coefficient was estimated to determine the performance of the kits using SPSS statistics software (IBM, version 20.0.0.2, Armonk, NY, USA) [7].

## 3. Results

### 3.1. RT-qPCR Results

Among the 326 prospective COVID-19 patients, 209 (64.10%) were diagnosed positive and the remaining 117 (35.89%) were negative by the reference RT-PCR method. The demographic data can be found in our previous rapid test study [7]. 

### 3.2. Performance of Aridia^®^ COVID-19 Real-Time PCR Test

The Aridia^®^ COVID-19 kit gave similar results to the reference method. Thus, the sensitivity and specificity of the Aridia^®^ COVID-19 kit were 100% in both cases (95% CI: 98.3% to 100.0% for sensitivity and 96.9% to 100.0% for specificity), compared to the reference method. The performance has been described in Table 1.

### 3.3. Performance of Allplex^TM^ 2019-nCoV Assay

The Allplex^TM^ 2019-nCoV assay kit could detect 205 out of 209 positive samples. For the 117 negative samples, Allplex^TM^ successfully detected all of them as negative. Comparing this result to that of reference RT-PCR, the sensitivity and specificity of the Allplex^TM^ kit were 98.1% (95% CI: 95.2% to 99.5%) and 100% (95% CI: 96.9% to 100%) respectively. These have been described in brief in Table 1.

### 3.4. Performance of Novel Coronavirus (2019-nCoV) Nucleic Acid Detection Kit, Sansure Biotech

Among the 209 positive samples, Sansure Kit could detect 206 samples. On the other hand, 112 out of 117 negative samples were detected as negative by the Sansure kit. The kit falsely amplified the target gene of five negative samples. These false positive and false negative values resulted in the reduced specificity of the kit (95.7%, 95% CI: 90.3% to 98.6%). The sensitivity (98.6%; 95% CI: 95.9% to 99.7%) was similar to the Allplex^TM^ kit, and lower than the Aridia^®^ COVID-19 kit (Table 1).

### 3.5. Sensitivity in Detection of N Gene

**Figure 1:**
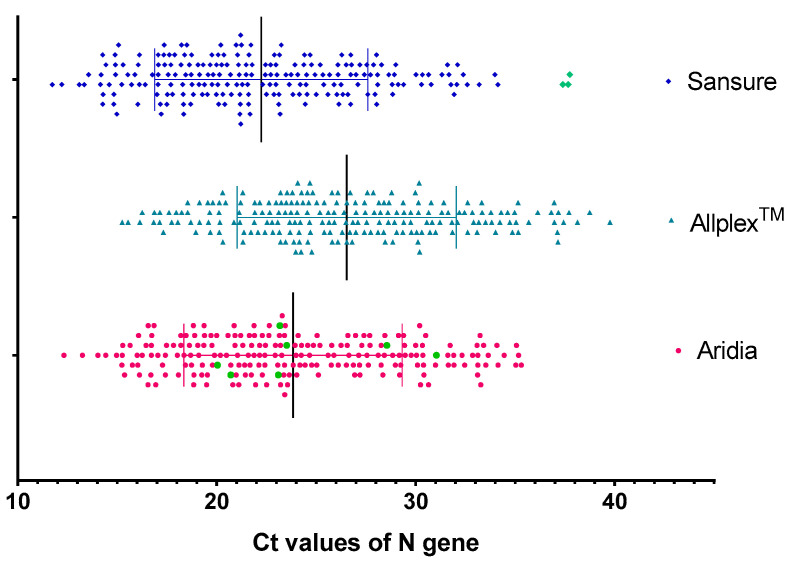
Distribution of Ct values (N gene). The green symbols in Aridia^®^ Kit depict the samples which were positive for Aridia^®^ only. The green symbols in Sansure Kit depict three falsely amplified N gene on negative samples.

The N gene was a common target of all three kits but their sensitivity of detection was different. The distribution of Ct values of the N gene is summarized in Table 2 and illustrated in Figure 1. 

**Figure 2:**
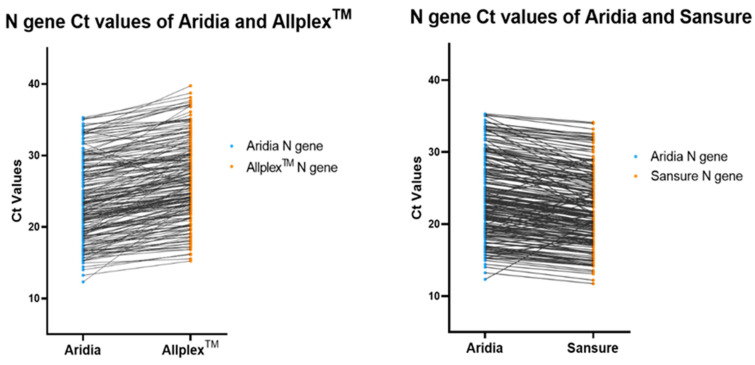
Line Graph comparing the Ct values of N gene for each sample, generated by different kits.

The Ct values of the N gene were slightly higher for the Allplex^TM^ kits than the Aridia^®^ kits but for the Sansure kits, they were mostly lower for the corresponding samples (*p* < 0.001). These have been shown in Figure 2 with a line graph indicating the Ct values of each sample by the terminal of each line.

**Figure 3:**
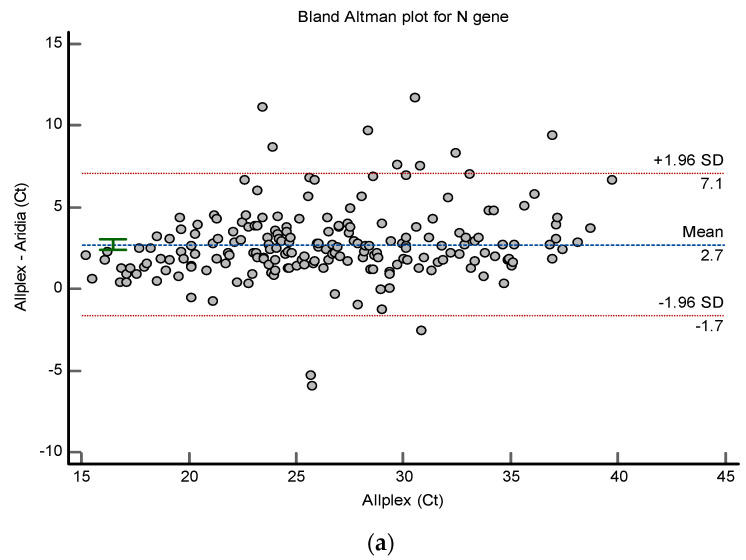

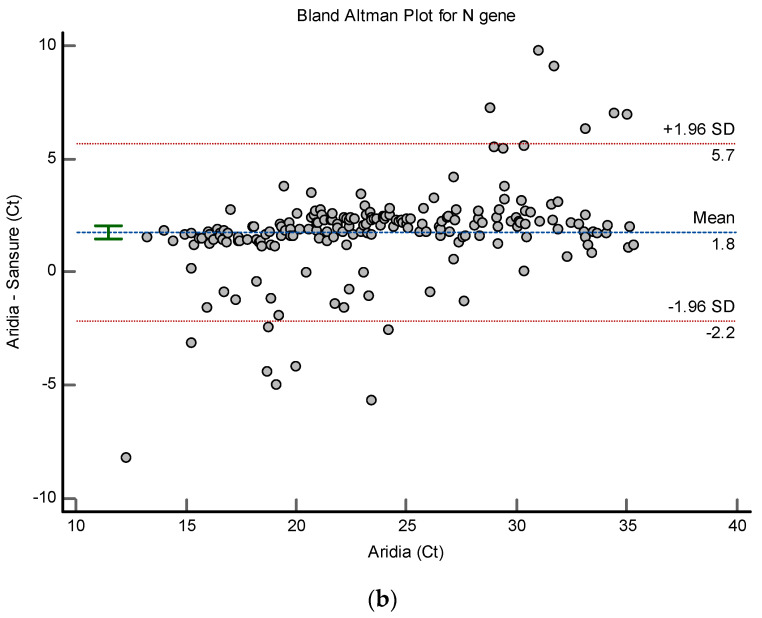
(**a**) Bland–Altman Plot of Ct values of N gene for AlplexTM-Aridia^®^ kits. The X-axis contains Ct values of Aridia^®^ kit and the Y axis contains the difference of Ct (Allplex^TM^ kit)-Ct (Aridia^®^ kit); (**b**) Bland–Altman Plot of Ct values of N gene for Aridia^®^-Sansure kits. The X-axis contains Ct values of Aridia^®^ kit and the Y axis contains the difference of Ct (Aridia^®^ kit)-Ct (Sansure kit).

The difference in Ct values has been also portrayed through the Bland–Altman plot in Figure 3a,b for the N gene of different kits. The analysis shows a significant mean difference of Ct values for Allplex^TM^-Aridia^®^ is 2.7 and 1.8 for Aridia^®^-Sansure (*p* < 0.001).

### 3.6. Sensitivity in Detection of E Gene

**Figure 4:**
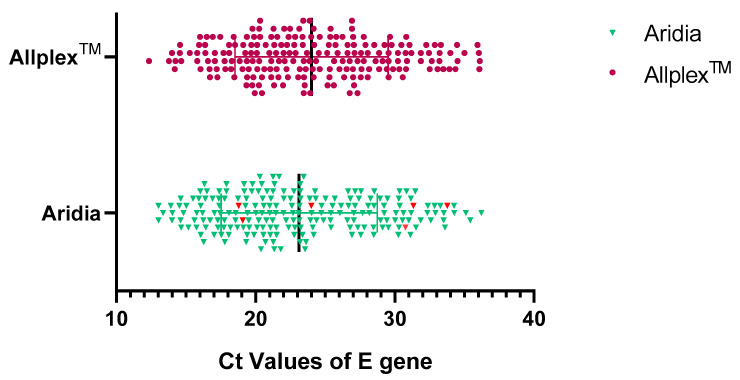
Distribution of Ct values (E gene). The red symbols in Aridia^®^ Kit depict the E gene of samples that were positive for Aridia^®^ kit only.

E gene was a common target of the Aridia^®^ and Allplex^TM^ kits but their sensitivity of detection was different. The E gene of six positive samples could not be amplified by Allplex^TM^ kits. Ct values were significantly different from each other (*p* < 0.0001). The distribution of Ct values of the E gene is summarized in Table 3 and illustrated in Figure 4.

### 3.7. Sensitivity in Detection of ORF1ab Gene

**Figure 5:**
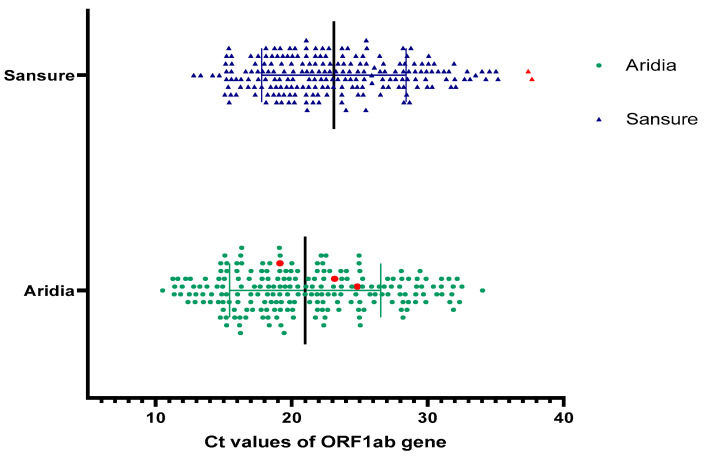
Distribution of Ct values (ORF1ab gene). The red circles for the Aridia^®^ plot depict the ORF1ab gene of samples that were positive for the Aridia^®^ kit only. Two red triangles for Sansure kit represent the false positive result.

The ORF1ab gene was a common target of the Aridia^®^ and Sansure kits but their sensitivity of detection was different. The ORF1ab gene of one sample could not be amplified by the Sansure kit. The distribution of Ct values of the ORF1ab gene is summarized in Table 4 and illustrated in Figure 5.

Additionally, the ORF1ab gene of two negative samples was falsely amplified by the Sansure kit with Ct > 35 which was shown in red triangles in Figure 5.

### 3.8. LLOD, Linearity, and Reproducibility

**Figure 6:**
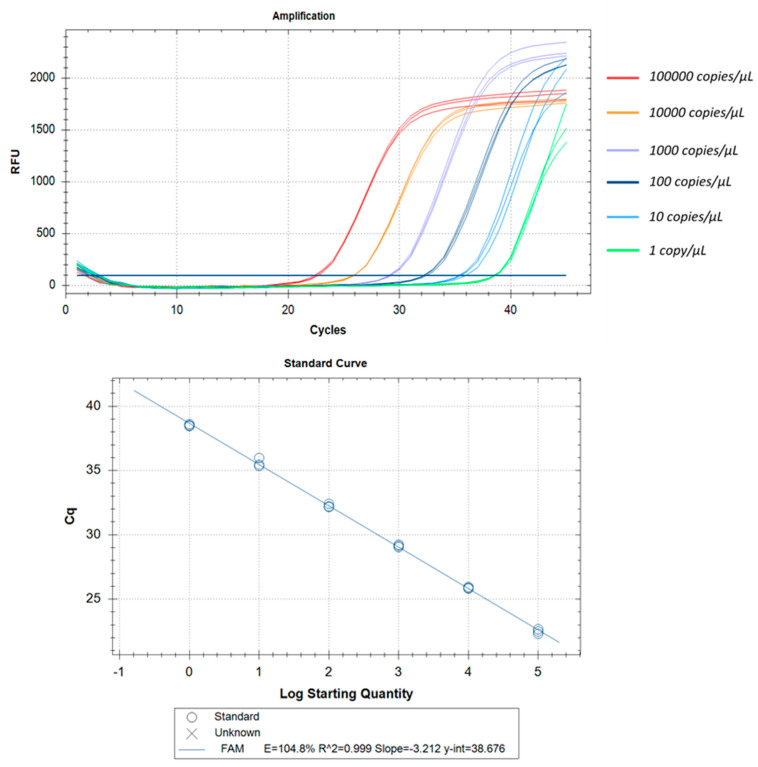
Analytical performance and LOD of the Aridia^®^ real-time RT-PCR. Plasmid control was diluted from 100,000 to 1 copy/µL. Amplification curves are represented by different colors for each different viral load. The standard curve has been made by a linear regression curve fit analysis (R^2^ = 0.999, E = 104.8%).

**Figure 7:**
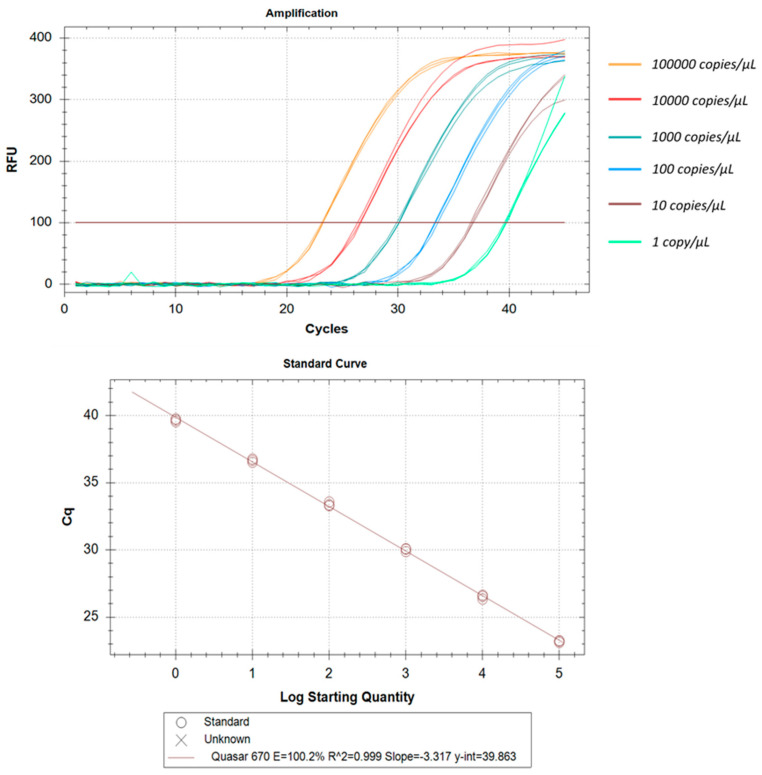
Analytical performance and LOD of the Allplex^TM^ real-time RT-PCR. Plasmid control was diluted from 100,000 to 01 copy/µL. Amplification curves are shown by different colors for each different viral load. The standard curve has been made by a linear regression curve fit analysis (R^2^ = 0.999, E = 100.2%).

**Figure 8:**
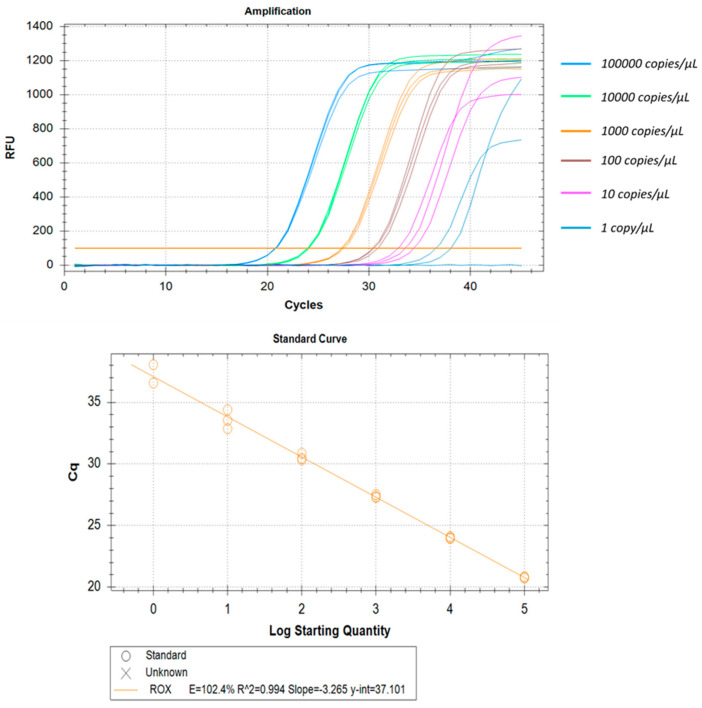
Analytical performance and LOD of the Sansure real-time RT-PCR. Plasmid control was diluted from 100,000 to 01 copy/µL. Amplification curves are portrayed by different colors for each different viral load. The standard curve has been made by a linear regression curve fit analysis (R^2^ = 0.994, E = 102.4%).

All three real-time RT-PCR kits evaluated in this study could detect as low as 1 copy/µL. Some of these kits could even detect even lower copy numbers but, due to lack of reproducibility, we have found the lower limit of detection as 1 copy/µL. The correlation coefficients (R^2^) were ≥0.994 and efficiency was around 100% in all cases. The intra-assay CV of Ct values for Aridia^®^, Allplex^TM^, and Sansure ranged from 0.17% to 0.97%, 0.26% to 0.54% and 0.31% to 2.82% respectively over six different concentrations. In the case of the Sansure kit, increased variations were observed in low copy numbers. Assay reproducibility was assessed through inter-assay Ct value variation for the same concentration level in two different independent triplicate runs. The CV for inter-assay fluctuated from 0.8% to 1.28%, 0.02% to 0.63% and 0.01% to 1.17% for Aridia^®^, Allplex^TM^, and Sansure respectively. The CV values point out the excellent reproducibility of all three assay methods. The LLOD, linearity, repeatability and reproducibility have been summarized in Table 5, Table 6 and Table 7 and portrayed in Figure 6, Figure 7 and Figure 8.

## 4. Discussion

A number of studies have been done so far to assess the performance of commercially available COVID-19 RT-PCR kits, but there are many kits being used for regular diagnosis that warrant further evaluation as well. Of the kits we evaluated in this study, CTK’s Aridia^®^ Kit was the most convenient compared to the two other kits, as it was an all-in-one master mix in ambient-stable lyophilized form, easy to prepare with the provided nuclease-free water and controls for each assay. The lyophilized format, also eases shipment and storage at room temperature. On the other hand, Allplex^TM^ kit and Sansure kits contain several vials and the contents are in liquid form, which require maintenance of the cold chain (−20 °C to 4 °C) and if the cold chain is interrupted because of logistic issues, the performance of the kits will be compromised.

Although several studies have already evaluated the Allplex^TM^ kit and Sansure Kit [15,17,18,19], none has been undertaken so far for the Aridia^®^ kit. Our study evaluated and compared the performance of the Aridia^®^ kit with already established and available commercial kits that are also highly used in this region, Allplex^TM^ kit and Sansure kit. The Aridia^®^ kit result showed the best performance among the three kits, with a sensitivity and specificity of 100%. The Allplex^TM^ kit’s specificity was also 100%, but it showed higher Ct values for the detection of the N gene (*p* < 0.001) than the other two kits. The performance of the Sansure Kit in this study fell slightly behind, as it failed to detect three positive and five negative samples correctly.

Compared to our study, a recent study evaluated three PCR kits, namely, GeneFinderTM (OSANG Healthcare Co., Seongnam, Korea), Sansure Biotech (Sansure Biotech Inc., Changsha, China), and TaqPathTM (Thermo Fisher, Waltham, MA, USA) on 354 randomly selected samples. They showed that the Sansure Biotech assay had better diagnostic performance than the GeneFinderTM and TaqPathTM PCR kits [20]. The Sansure kit was evaluated against the FDA EUA 2019-nCoV CDC kit (IDT, Coralville, IA) and showed 95.3% of sensitivity with a limit of detection as low as 1000 copies/mL in South America [15]. In another study, the Sansure kit had a low sensitivity of 83.3% although a specificity of 100% in a very limited number of samples in a study conducted in Guangxi, China [21]. For routine COVID-19 real-time RT-PCR tests, the Sansure kit had a 90% of positive percent agreement in 86 symptomatic patients in Malaysia [22]. The Sansure kit performs well in samples Ct < 30 with a sensitivity of 94.6% even for extraction-free SARS-CoV-2 assay but the overall sensitivity falls to 69.6% in a sample size of 94 patients with 69 positives. Compared to that, the Allplex^TM^ had a sensitivity of 98.7% in a study with 115 positive samples when NGS was used as a reference in Malaysia [19]. Another study assessed the performance of the Allplex^TM^ in more than 1000 samples including extracted RNA, nasopharyngeal swabs, and saline gargles using the whole genome sequencing process as standard. This kit could easily detect several mutations with 100% sensitivity [18]. The performance can drastically fall to 74% of sensitivity for the extraction-free assay method [23].

In our study, we found the LLOD for all kits to be the same, 1 copy/µL and hence, a comparative analysis could not be drawn among these kits for this parameter. A notable major limitation was the multiple freeze-thaw of the samples. The reference RT-PCR assay was performed within 24 h of sample collection, with the maintenance of the cold chain. Due to limitations of resources and logistics, these kits were evaluated later, with archived extracted RNA that has been thawed multiple times. Another drawback of the study is that we did not have any choice in sample collection, especially with higher Ct values or patients in critical hospital settings for better comparison of the kits as we used the diagnostic facilities only. Additionally, these are commercial kits and hence, they only mentioned their gene target but did not disclose the sequences. Their varied performance can be due to different gene targets than the reference one. The primer can also play a role in better detection but we could not analyze the performance in terms of primer sequences. Nevertheless, these kits performed very well in terms of sensitivity and specificity. These kits are only used for diagnostic purposes, and other studies are required to evaluate these kits’ ability to detect all the variants of concerns and point mutations that are emerging worldwide.

## 5. Conclusions

Many countries are already using rapid tests for the diagnosis of COVID-19 in clinical settings which require further confirmation for negative tests in highly symptomatic patients. There is no better alternative to RT-PCR tests, and they are still performed in hospitals extensively. Therefore, good-quality kits are required so that no patient remains misdiagnosed. From the detailed investigation in our study, we conclude that the Aridia^®^ kit showed the best performance of all the three kits evaluated, followed by the other two commercially available RT-PCR kits included in this study, and thus, can be used for the routine molecular diagnosis of COVID-19.

## Figures and Tables

**Table 1:** Performance analysis of Aridia^®^, Allplex^TM^ and Sansure COVID-19 RT-PCR kits with CDC RT-PCR as reference.

Parameter	*Aridia^®^*	Allplex^TM^	Sansure
Sensitivity	100 (95% CI: 98.3% to 100%)	98.1% (95% CI: 95.2% to 99.5% )	98.6% (95% CI: 95.9% to 99.7%)
Specificity	100.00% (95% CI: 96.9% to 100%)	100% (95% CI: 96.9% to 100%)	95.7% (95% CI: 90.3% to 98.6%)
AUC (Area Under Curve)	1 (95% CI: 0.99 to 1.0)	0.99 (95% CI: 0.97 to 0.99)	0.971 (95% CI: 0.95 to 0.99)
Positive Predictive Value	100 %	100.00%	97.6% (95% CI: 94.6% to 98.9%)
Negative Predictive Value	100 %	99.90% (95% CI: 99.7% to 99.9%)	99.9% (95% CI: 99.8% to 99.9%)
Accuracy	100.00% (98.9% to 100%)	99.90% (95% CI: 98.7% to 100%)	95.87% (95% CI: 93.1% to 97.8%)
κ	1.0	0.97	0.95

**Table 2:** Distribution of Ct values of N gene in different ranges.

Ct Values	*Aridia* ^®^	Allplex^TM^	Sansure
<25	128 (61.2%)	89 (43.6%)	147 (70.3%)
25–30	44 (21.2%)	59 (28.9%)	42 (20.1%)
30–35	33 (15.8%)	40 (19.6%)	17 (8.1%)
>35	4 (1.9%)	16 (7.8%)	0 (0%)
Total samples	209	204 **	206

** 204 samples were false negative and Allplex^TM^ kit could not amplify N gene of one sample.

**Table 3:** Distribution of Ct values of E gene in different ranges.

Ct Values	*Aridia*^®^ Kit, n (%)	Allplex^TM^ Kit, n (%)
<25	136 (65.1%)	117 (57.6%)
25–30	39 (18.7%)	53 (26.1%)
30–35	31 (14.8%)	28 (13.8%)
>35	03 (1.4%)	05 (2.5%)
Total samples	209	203 ***

*** E gene was unamplified in six samples.

**Table 4:** Distribution of Ct values of ORF1ab gene in different ranges.

Ct Values	*Aridia*^®^ Kit, n (%)	Sansure Kit, n (%)
<25	157 (75.1%)	137 (65.9%)
25–30	38 (18.2%)	47 (22.6%)
30–35	14 (6.7%)	20 (9.6%)
>35	00 (0%)	04 (1.9%)
Total samples	209	208 ****

**** ORF1ab gene was unamplified in one sample and amplified in two false positive samples with Ct value > 35.

**Table 5:** Repeatability and reproducibility of real-time RT-PCR assay for Aridia^®^ kit.

Copies/µL	Intra Assay Variation of Ct Values						Inter Assay Variation of Ct Values				
	Replicate 1	Replicate 2	Replicate 3	Mean	SD	CV (%)	Assay 1	Assay 2	Mean	SD	CV (%)
100,000	22.53	22.38	22.7	22.54	0.16	0.71	22.54	22.79	22.67	0.18	0.80
10,000	25.88	25.99	25.91	25.93	0.06	0.22	25.93	25.46	25.70	0.33	1.28
1000	29.23	29.17	29.06	29.15	0.09	0.30	29.15	28.57	28.86	0.41	1.44
100	32.31	32.46	32.2	32.32	0.13	0.40	32.32	31.90	32.11	0.30	0.92
10	36.05	35.52	35.4	35.66	0.35	0.97	35.66	35.21	35.43	0.32	0.89
1	38.65	38.54	38.53	38.57	0.07	0.17	38.57	39.04	38.81	0.33	0.85

**Table 6:** Repeatability and reproducibility of real-time RT-PCR assay for Allplex^TM^ kit.

Copies/µL	Intra Assay Variation of Ct Values						Inter Assay Variation of Ct Values				
	Replicate 1	Replicate 2	Replicate 3	Mean	SD	CV (%)	Assay 1	Assay 2	Mean	SD	CV (%)
100,000	23.22	23.13	23.26	23.20	0.07	0.29	23.20	23.18	23.19	0.02	0.07
10,000	26.63	26.35	26.55	26.51	0.14	0.54	26.51	26.49	26.50	0.02	0.06
1000	30.1	30.06	29.88	30.01	0.12	0.39	30.01	30.01	30.01	0.00	0.02
100	33.56	33.35	33.33	33.41	0.13	0.38	33.41	33.34	33.38	0.05	0.16
10	36.52	36.76	36.66	36.65	0.12	0.33	36.65	36.59	36.62	0.04	0.10
1	39.66	39.55	39.76	39.66	0.11	0.26	39.66	39.30	39.48	0.25	0.63

**Table 7:** Repeatability and reproducibility of real-time RT-PCR assay for Sansure kit.

Copies/µL	Intra ASSAY Variation of Ct Values						Inter Assay Variation of Ct Values				
	Replicate 1	Replicate 2	Replicate 3	Mean	SD	CV (%)	Assay 1	Assay 2	Mean	SD	CV (%)
100,000	20.86	20.76	20.74	20.79	0.06	0.31	20.79	20.68	20.74	0.07	0.35
10,000	24.1	23.95	24	24.02	0.08	0.32	24.02	24.00	24.01	0.01	0.04
1000	27.52	27.36	27.28	27.39	0.12	0.45	27.39	27.24	27.31	0.11	0.39
100	30.87	30.47	30.35	30.56	0.27	0.89	30.56	30.53	30.55	0.02	0.07
10	34.4	32.87	33.56	33.61	0.77	2.28	33.61	33.62	33.61	0.00	0.01
1	36.58	--	38.07	37.33	1.05	2.82	37.33	36.71	37.02	0.43	1.17

## Data Availability

Not applicable.
